# Time-out under scrutiny: examining the relationships among the discipline strategy time-out, child well-being and attachment and exposure to adversity

**DOI:** 10.1192/bjp.2024.228

**Published:** 2025-08

**Authors:** Alex Roach, Rebecca McLean, Antonio Mendoza Diaz, David Hawes, Mark Dadds

**Affiliations:** School of Psychology, University of Sydney, Sydney, Australia; Discipline of Psychiatry & Mental Health, School of Clinical Medicine, University of New South Wales, Sydney, Australia; Tasmanian Centre for Mental Health Service Innovation, Tasmanian Health Service, Tasmania, Australia

**Keywords:** Child and adolescent psychiatry, conduct disorders, psychological treatments, trauma and stressor-related disorders, mental health services

## Abstract

**Background:**

Discipline is a crucial aspect of parenting, shaping child development and behaviour. Time-out, a widely used disciplinary strategy with a strong evidence-base, has recently come under scrutiny with concerns about potential adverse effects on children's emotional development and attachment, particularly for those with a history of adversity.

**Aims:**

To contribute critical empirical insights to the current controversy surrounding time-out by exploring the associations among time-out implementation, parent–child attachment and child mental health, and whether adversity exposure moderated these associations.

**Method:**

This cross-sectional study utilised a nationally representative sample of 474 primary caregivers in Australia, with children aged 6–8 years, who completed an online survey. Measures included the Implementation of Time-out Scale, Adverse Life Experiences Scale, Primary Attachment Style Questionnaire, Strengths and Difficulties Questionnaire and Spence Child Anxiety Scale.

**Results:**

Appropriately implemented time-out was associated with enhanced mental health and attachment, while inappropriate time-out correlated with adverse child outcomes. Exposure to adversity moderated the relationship between time-out implementation and child well-being, such that children exposed to adversity were most likely to experience attachment enhancement from appropriately implemented time-out.

**Conclusions:**

Despite recent concerns of harm caused by time-out, particularly for children with a history of adversity, findings support the beneficial impact of time-out on child well-being and attachment when implemented in accordance with evidence-based parameters. Combatting misinformation and disseminating evidence-based time-out guidelines is crucial for promoting child well-being and attachment, especially for children who have experienced adversity.

Discipline is a fundamental component of parenting that teaches children appropriate behaviour and emotion regulation skills, thereby reducing their vulnerability to future societal rejection and mental health disorders.^1^ Among the myriad of disciplinary strategies employed by parents and caregivers, time-out has emerged as one of the most utilised and effective evidence-based approaches.^2–6^ However, recent years have witnessed a growing rejection of time-out as a disciplinary tool,^7^ driven by concerns related to implementation drift^8^ and a wave of misinformation surrounding its efficacy and potential adverse effects.^9,10^ The use of time-out with children who have a history of adverse childhood experiences (ACEs) has become particularly contentious.^1,11^ Such children are at an increased risk of exhibiting behavioural issues^12^ and are especially in need of effective parenting strategies for optimal recovery. In the trauma context, there are concerns that time-out may adversely affect attachment bonds, hinder emotional development and even result in re-traumatisation.^1,11^

While proponents of time-out emphasise that the discipline strategy has potential to enhance child well-being and attachment when implemented appropriately,^1,13,14^ opponents argue that regardless of implementation, time-out may have detrimental consequences on children's emotional and psychological well-being.^9^ Research on the implementation of time-out in the community has primarily centred on parents’ perspectives towards the disciplinary strategy and their adherence to evidence-based parameters.^3,7,8^ However, there has been a notable gap in assessing the impact of variations in implementation on child well-being and attachment bonds. This cross-sectional study aims to address this gap, by investigating the associations among time-out implementation, parent–child attachment and child mental health, and test the appropriateness of time-out for children with a history of adversity.

## Method

### Participants and procedure

A total of 512 primary caregivers participated in the anonymous online survey recruited through the independent survey platform, Qualtrics. Participants were primary caregivers of a child aged 6–8 years, residing in Australia, with access to a computer and skill in computer literacy. Parents with more than one child answered questions about their eldest child. The sampling frame was stratified by region and primary caregiver gender to be nationally representative.^15^ Before beginning the survey, participants reviewed a participant information statement and provided written informed consent. The participants then completed the 30-min survey, following which they were provided with parenting resources. Data quality checks eliminated 38 invalid responses (e.g. speeding, random responding or duplicate responses) resulting in a final analytic sample of 474 participants, which equates to 0.05% of the total eligible population of parents in Australia.^16^ Table 1 outlines the demographic characteristics of the sample.

**Table 1:** Demographic characteristics of participants (*N* = 474)

Characteristic	Category	*n*	(%)
Child's gender	Female	234	49.4
	Male	240	50.6
Child's age	6	126	26.6
	7	192	40.5
	8	156	32.9
Participant's relationship to child	Mother (biological)	226	47.7
	Father (biological)	235	49.6
	Mother (non-biological)	4	0.8
	Father (non-biological)	3	0.6
	Other primary caregiver	6	1.3
Participant's marital status	Sole caregiver	89	18.8
	Married/de facto	350	73.8
	Divorced/separated	28	5.9
Caregiver's age (years)	18–20	3	0.6
	21–29	42	8.9
	30–39	251	53.0
	40–49	155	32.7
	50–59	17	3.5
	60 or older	6	1.3
Caregiver's level of education	Secondary school (Year 10)	19	4.0
	Secondary school (Year 12)	49	10.3
	TAFE/diploma/professional certificate	126	26.6
	Undergraduate degree	153	32.3
	Master's degree, PhD or other postgraduate degree	127	26.8
Residential state	Australian Capital Territory	12	2.5
	New South Wales	154	32.5
	Northern Territory	0	0
	Victoria	129	27.2
	Queensland	95	20.1
	South Australia	32	6.8
	Western Australia	41	8.6
	Tasmania	11	2.3

TAFE, technical and further education.

The authors assert that all procedures contributing to this work comply with the ethical standards of the relevant national and institutional committees on human experimentation and with the Helsinki Declaration of 1975, as revised in 2013. All procedures involving human participants were approved by University of Sydney Human Ethics Committee (2020/178). The survey was conducted from May to July 2020.

### Measures (in order of survey presentation)

#### Time-out use and implementation

The Implementation of Time-out Scale^17^ is a ten-item parent self-report measure assessing the appropriateness of time-out implementation in accordance with evidence-based parameters.^3,8^ Items assessed were as follows: time-out frequency, behavioural context (e.g. owing to aggression, owing to anxiety) duration, location and escape contingencies. Responses were measured primarily on a 5-point Likert scale ranging from *Never* to *Always*. Items included the following: ‘I give my child time-out if he/she makes a mistake’ and ‘My child can see or play with books, toys, TV or similar things during time-out’.

#### Parent–child attachment

The Primary Attachment Style Questionnaire (PASQ)^18^ was adapted to measure the quality of a child's attachment to their primary caregiver. The PASQ has good predictive validity and acceptable reliability for gauging attachment styles in children.^18^ Relevant items from the longer measure with the highest factor loadings were selected for inclusion, resulting in ten items with a 5-point Likert scale reflecting secure, ambivalent and avoidant attachment scales. Scores were summed and divided by the number of relevant items to provide an average for each attachment style, following which the ambivalent and avoidant attachment styles were collapsed into one insecure attachment scale. For parsimony, insecure attachment only was included in the final analysis being the most relevant to the study aims of investigating the potential harms of time-out. Items included the following: ‘If my child tries to discuss things with me, they end up feeling angry and frustrated’ and ‘I don't like demonstrations of affection from my child, physical or otherwise’.

#### Parenting and family functioning

The Parenting and Family Adjustment Scales^19^ is a 30-item measure assessing various aspects of parenting and family functioning. The scale includes seven domains: parental consistency, coercive parenting, positive encouragement, parent–child relationship, parental adjustment, family relationships and parental teamwork. The caregivers rate each item on a scale from 0 (*not true of me at all*) to 3 (*true of me very much, or most of the time*), with higher scores indicating higher levels of dysfunctional parenting. Items included ‘I give my child attention (e.g. a hug, wink, smile or kiss) when they behave well’ and ‘I shout or get angry with my child when they misbehave’. Please note that the Parental Teamwork Scale was omitted from the analysis because of the significant representation of solo parents within the sample.

#### Adverse childhood experiences

The Adverse Life Experiences Scale (ALES)^12^ is 23-item measure of lifetime experience demonstrating good reliability and validity in community samples. The ALES was developed as an extension of the original ten-item ACE survey^20^ to capture a broader range of adverse experiences (e.g. peer victimisation, minority adversity). Parents endorsed items on a dichotomous (yes/no) scale and they were summed to provide a total score reflecting the number of adversities the child had been exposed to. Items included ‘Has your child lived with someone who misused drugs or alcohol?’ and ‘Has an adult repeatedly sworn at, insulted, put down, humiliated, or threatened to hurt your child?’.

#### Trauma symptoms

In addition to capturing exposure to adverse events, symptoms of trauma were measured through a subscale of the Spence Child Anxiety Scale,^21^ which includes six items assessing symptoms of post-traumatic stress disorder (PTSD). Participants reported the presence of PTSD symptoms in their child following exposure to a very stressful event on a 5-point Likert scale ranging from *Very often true* to *Not true at*. Items included ‘Has bad dreams or nightmares about the event’, ‘Remembers the event and becomes distressed’ and ‘Shows bodily signs of fear (e.g. sweating, shaking or racing heart) when reminded of the event’.

#### Child emotional and behavioural symptoms

The Strengths and Difficulties Questionnaire (SDQ)^22^ is a 25-item parent-report questionnaire that measures four main domains of child psychopathology: emotional symptoms, conduct problems, hyperactivity-inattention and peer problems. The widely used measure has demonstrated good validity and internal reliability^23,24^ and sound psychometric properties for use in large population studies.^22^ Using a 3-point Likert scale ranging from *Not true* to *Certainly true*, participants indicated how much the target item applied to their child. Items included ‘Considerate of other people's feelings’ and ‘Restless, overactive, cannot stay still for long’.

### Data analysis

Sample differences were assessed with the χ^2^-test for categorical variables and one-way analysis of variance for continuous variables. Linear regression models examined the associations of time-out implementation, exposure to adversity and child well-being. A linear model with SDQ scores included as the dependent variable identified all other parenting factors (e.g. positive encouragement, coerciveness) as significant predictor variables. These parenting variables were included as a second regression model to ascertain that they were not confounding the primary associations under investigation. The main analysis models were computed in a stepwise hierarchical manner, where exposure to adversity and overall time-out use were entered in the first model, covariates added to the second model and an interaction term (Time-out × ACE) included in the third model (correlations table in supplementary materials). Tests to assess the assumption of collinearity indicated that multicollinearity was not a concern (i.e. variance inflation factor < 5 for all variables).^25^ Simple slope analyses were conducted to investigate significant regression interaction terms. Associations are expressed by the unstandardised regression coefficients (*B*) and the 95% confidence intervals, and *P*-values <0.05 were considered statistically significant for two-sided tests. All analyses were conducted with SPSS software, Windows version 26.0.0.0.^26^

## Results

### Characteristics of participants

Table 1 details participant sociodemographic factors. Preliminary analyses revealed that no demographic variables systematically related to child outcomes. When benchmarked against Australian norms for child behaviour and adversity,^12^ the sample demonstrated comparative rates of exposure to adversity and slightly worse emotional and behavioural symptoms.^27^ Consistent with previous research, most parents (72.4%) were found to have used some form of time-out with their children^3,6,7^ and a large proportion of these parents were found to implement time-out in a manner that was not consistent with evidenced-based parameters.^8^ For example, around one third of parents (33.1%) had used time-out with their child when they were anxious and almost half of parents (49.8%) used inappropriate time-out locations with distractions or toys.

### Effect of ACEs, time-out and other parenting factors in predicting externalising symptoms

Model 1 revealed that greater exposure to adversity was associated with increased externalising problems (*B* = 0.445, 95% CI 0.324–0.565, *P* < 0.001; Supplementary material, Table 1, available at https://doi.org/10.1192/bjp.2024.228), while time-out implementation was associated with reduced externalising problems (*B* = −0.140, 95% CI −0.198 to −0.082, *P* < 0.001). After entering other parenting covariates and the interaction term (Model 3) into the analysis, the association between time-out and externalising symptoms was no longer observed. Increases in adversity exposure (*B* = 0.316, 95% CI −0.014 to 0.115, *P* < 0.001), harsh and coercive parenting (*B* = 0.474, 95% CI −0.270 to 0.142, *P* < 0.001) and poor parent–child relationships (*B* = 0.196, 95% CI −0.004 to 0.301, *P* = 0.012) were associated with higher externalising scores. Moderating effects from the variables time-out implementation, adversity exposure and externalising symptoms were tested and no significant interaction was observed.

### Effect of ACEs, time-out and other parenting factors in predicting internalising symptoms

Model 1 found that greater exposure to adversity was associated with increased internalising problems (*B* = 0.456, 95% CI 0.346–0.566, *P* < 0.001), while appropriate time-out implementation was associated with reduced internalising problems (*B* = −0.263, 95% CI −0.316 to −0.210, *P* < 0.001). When other parenting factors and the interaction term were added to the model (Model 3), several other parenting variables were also found to be significantly associated with internalising symptoms (Supplementary material, Table 2), and an interaction between adversity exposure and time-out implementation was observed.

**Fig. 1:**
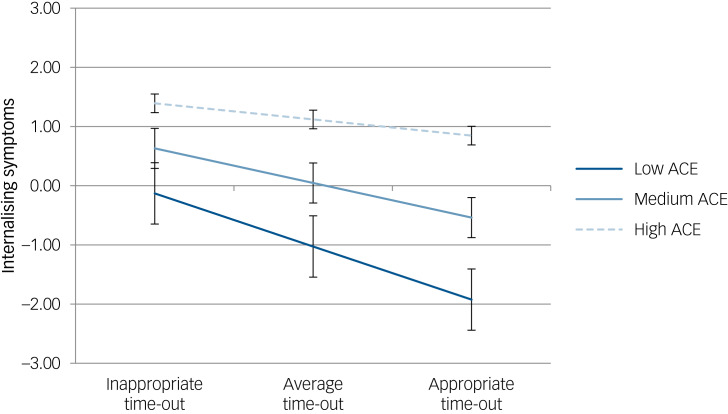
Plot of internalising symptoms on time-out at varying levels of adversity. The low adverse childhood experiences (ACE) slope was significant, while the high ACE slope was not significant.

Simple slope analyses tested the significance of the moderating interaction (Fig. 1; Supplementary material, Table 3). Appropriate and inappropriate levels of time-out implementation and high and low adversity exposure were operationalised as one standard deviation above or below the mean. For children with low levels of adversity exposure, increasing the appropriateness of time-out implementation was associated with reductions in internalising symptoms (*B* = −0.153, 95% CI −0.227 to −0.079, *P* = <0.001). An association between time-out implementation and internalising symptoms was not observed in the high ACE group, suggesting that time-out implementation was less strongly associated with internalising symptoms for children with high ACE exposure.

### Effect of ACEs, time-out and other parenting factors in predicting parent–child attachment

Model 1 demonstrated a positive association between exposure to adversity and insecure attachment (*B* = 0.030, 95% CI 0.004–0.056, *P* = 0.023); however, this association was not sustained in Model 3 after other parenting factors and the interaction term were added to the model (Supplementary material, Table 4). Effective implementation of time-out was associated with improvements in insecure attachment (*B* = −0.098, 95% CI −0.110 to −0.086, *P* < 0.001), and after other parenting factors and the interaction term were added to the model (Model 3), several other parenting variables were also found to have significant associations with insecure attachment. Notably, an interaction effect between adversity exposure and time-out implementation was identified (*B* = −0.003, 95% CI −0.006 to 0.000, *P* = 0.043).

**Fig. 2:**
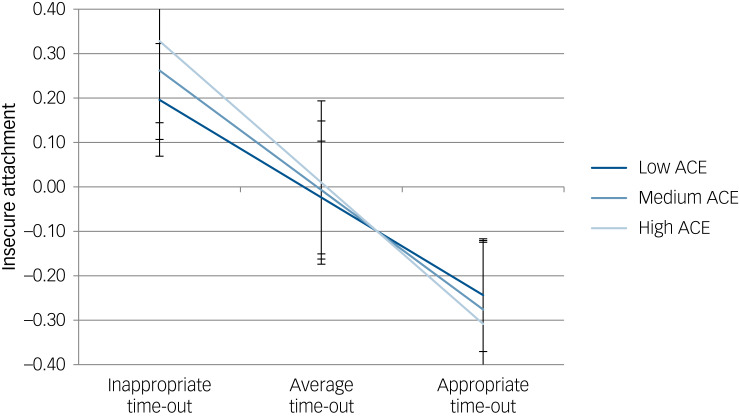
Plot of insecure attachment on time-out at varying levels of adversity. The high and low adverse childhood experiences (ACE) slopes were found to be significant.

Simple slope analyses were performed to examine the direction of the interaction effect (Supplementary material, Table 5; Fig. 2). Results showed that level of appropriate time-out implementation was more strongly associated with insecure attachment for children with high ACE exposure (*B* = −0.054, 95% CI −0.069 to −0.039, *P* < 0.001) than for children with low ACE exposure (*B* = −0.038, 95% CI −0.055 to −0.021, *P* < 0.001).

### Effect of ACEs, time-out and other parenting factors in predicting trauma symptoms

A positive association between exposure to adversity and trauma symptoms (*B* = 0.094, 95% CI 0.073–0.114, *P* < 0.001) was found in Model 1 and this association was sustained in Model 3 after other parenting factors and the interaction term were added to the model (Supplementary material, Table 6). Effective implementation of time-out was associated with improvements in trauma symptoms (*B* = −0.029, 95% CI −0.039 to −0.019, *P* < 0.001); however, after other parenting factors and the interaction term were added to the model (Model 3), this association was no longer significant. Several other parenting variables were found to have significant associations with trauma symptoms and the interaction effect between adversity exposure and time-out implementation was found to be significant.

**Fig. 3:**
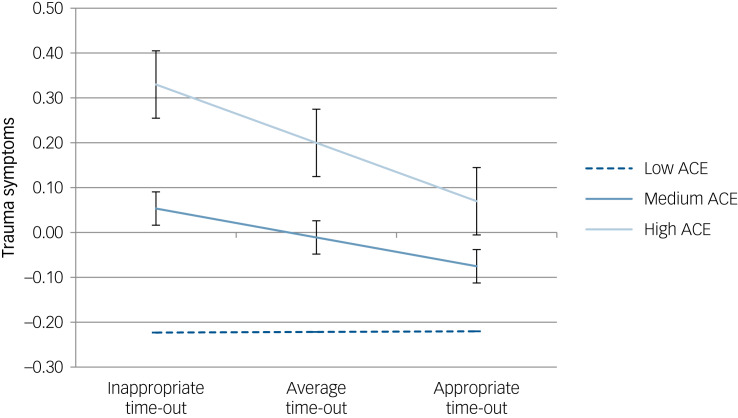
Plot of trauma symptoms on time-out at varying levels of adversity. The high adverse childhood experiences (ACE) slope was found to be significant, while the low ACE slope was not significant.

Simple slope analyses revealed that time-out implementation was more strongly associated with trauma symptoms for children with high ACE exposure (*B* = −0.026, 95% CI −0.039 to −0.013, *P* < 0.001) compared to children with low ACE exposure (*B* = −0.001, 95% CI −0.016 to 0.014, *P* = 0.885; Fig. 3; Supplementary material, Table 7).

## Discussion

### Main findings and interpretations

Results from a nationally representative survey found support for time-out as a disciplinary tool when implemented appropriately, even for children with previous adversity exposure. When time-out was implemented in accordance with evidence-based parameters, time-out was associated with reduced levels of child mental health issues and attachment problems. Conversely, when implementation was not aligned with evidence-based parameters (e.g. inconsistent, reinforcing, punitive), time-out was associated with detrimental child mental health and attachment outcomes. Importantly, when time-out is administered in a manner that lacks procedural fidelity (e.g. not administered in response to misbehaviour, executed with parental anger or hostility), this attempt at discipline is not considered time-out to experts in the field.^1^ For the purposes of this paper, however, we used a continuous scale to examine how varying degrees of appropriate time-out implementation affect child outcomes. Adversity exposure moderated the relationship between time-out implementation and child well-being, such that children exposed to adversity were most likely to experience attachment enhancement from appropriately implemented time-out, and attachment impairment when time-out was not implemented appropriately. Notably, this study did not find evidence that children with a history of adversity are ill-suited to time-out. Rather, when time-out is administered in a calm, consistent and safe manner, these children may derive an even greater benefit from such an approach to discipline that is predictable and models regulated emotions from the caregiver.

Appropriate time-out implementation was found to be associated with reductions in externalising symptoms. This finding is consistent with previous longitudinal studies showing that appropriate time-out implementation leads to reductions in behaviour problems.^2^ However, it may also suggest that adhering to evidence-based parameters for time-out is easier when children are better behaved. When all other parenting components were entered into the model, the effect of time-out implementation was no longer significant. This finding aligns with the understanding that time-out is not a standalone intervention but rather part of a broader spectrum of effective parenting strategies. When other factors such as positive reinforcement, strong attachment and the absence of coercive cycles are securely in place, the need for time-out naturally diminishes. By design, time-out serves as a temporary tool rather than a necessary long-term solution.^1^ Adversity exposure did not moderate the relationship between time-out and externalising symptoms, indicating that when implemented appropriately, time-out is effective in improving behaviour for children with and without adversity exposure. These results provide further support for the growing evidence-base supporting the use of parenting interventions that include time-out for children exposed to adversity.^4,28,29^

Interestingly, appropriate time-out implementation was associated with lower internalising symptoms. While previous systematic reviews of parent training programmes have found reduced comorbid anxiety symptoms post-treatment, this study is the first to find that time-out implementation itself, rather than a suite of positive parenting strategies, is associated with reduced internalising symptoms, despite time-out not targeting anxiety symptoms directly.^30^ While this result may indicate that time-out fidelity is easier to achieve with less anxious children, another possible mechanism of change could be the transdiagnostic improvement to emotion regulation that appropriate time-out encourages.^1^ Exposure to negative-feeling states and the opportunity to practice self-regulation before and during time-out may generalise to improvements in regulating internalising and externalising symptoms. Consistent with previous literature,^31^ children with high exposure to adversity had higher internalising symptoms, which were not associated with time-out implementation. These children may require more specific trauma-informed anxiety strategies to target internalising symptoms, compared to their low adversity exposure peers.

While a primary concern raised by opponents of time-out surrounds its impact on parent–child attachment, interestingly, the results of this study found that appropriate time-out implementation is associated with reduced attachment insecurity. As the direction of this association is not known, there are numerous possible hypotheses one can draw from this result. First, and as stated previously, it may be that more securely attached children facilitate conditions for more effective discipline. For example, securely attached children may not be as challenging when being put into time-out, may be more comfortable separating during time-out, leave time-out less often and be less emotionally reactive throughout the procedure, which aids in the parents’ ability to be more consistent and implement time-out appropriately. When considering alternate possible causal directions of results, recent re-conceptualisations of time-out through an attachment lens provide suggestions on how appropriately implemented time-out may positively influence the parent–child relationship.^1,32^ Specifically, appropriate time-out implementation may be associated with more secure parent–child relationships as it mirrors secure attachment processes, where time-out provides the conditions for successful separation and reunion during the procedure without threatening attachment bonds.^8^

We found that adversity moderates the association between time-out and insecure attachment, which may again reflect child factors that influence how effectively parents can implement time-out, but results may also suggest that children with high adversity exposure are especially responsive to both protective and detrimental effects of appropriate versus inappropriate time-out implementation. The heightened impact of inappropriate time-out on children with high adversity exposure aligns with research indicating that these children exhibit increased emotional reactivity to perceived threats.^31^ When administered in a harsh and punitive manner, time-out may be interpreted as a threat by these children, exacerbating attachment and trauma symptoms. Similarly, supportive caregiving is shown to be particularly protective for children who have experienced trauma, buffering against heightened threat sensitivity and mitigating adverse mental health outcomes.^31^ When time-out is administered appropriately, in a calm, consistent manner, children with previous adversity may benefit even more from this approach to discipline, which creates a sense of safety and predictability.

### Strengths, limitations and future directions

This study is the first to empirically examine concerns surrounding the time-out controversy regarding time-out implementation, attachment and trauma symptoms in a community sample of young children. Nationally representative general population data was used, avoiding selection biases associated with the use of convenience samples.^33^ However, it is important to note that the representative sample was limited by participants’ access to internet/technology and digital literacy skills. Further, many covariates were assessed, which allowed us to adjust for major confounders, including other parenting risk and protective factors.

The study has several limitations. First, the survey design relied on parental self-report, which can differ from children's reports of their symptomology^14,34^ and be hampered by social desirability reporting^35^ and self-serving bias.^36^ Second, in measuring adversity exposure, we combined a range of stressful events into a ‘childhood adversity’ measure, potentially compromising the findings’ generalisability owing to event heterogeneity. We also did not capture information on adversity timing, duration and chronicity, which may have influenced positive associations of improved trauma symptoms. Third, while insights into time-out implementation from a community sample of parents are valuable to consider from a population health standpoint, the clinical application of these findings is less clear. Lastly, the study's cross-sectional nature restricted conclusions, hindering the identification of causal mechanisms, direction of associations and causal inferences.

Despite these limitations, our findings provide empirical justification for further analysis exploring the relationship among time-out implementation, child mental health and adversity. Future research should utilise large-scale prospective longitudinal designs to investigate causal mechanisms and consider incorporating multi-informant data sources, such as parents, children and teachers, to enhance the reliability of childhood experience accounts. The impact of adversity exposure could be better understood by capturing data on timing, duration and chronicity of adversity, as well as caregivers’ own exposure to adversity, which is known to influence parenting. To comprehensively address the main concerns raised by time-out opponents, future research should prioritise recruiting clinical samples of children with behavioural issues and trauma backgrounds, improving the study findings’ applicability to clinical populations of interest.

### Clinical implications

This study contributes to the important discourse surrounding the time-out controversy and provides support for the appropriate use of time-out. Namely, our findings warn against the use of inappropriate time-out practices while highlighting the positive association that consistent, calm and safe discipline has with child well-being. To briefly recap, the current controversy surrounding the appropriateness of time-out was in large part catalysed by an influential article published in *Time* magazine titled ‘Time-outs are hurting your child’.^9^ Although initially critical of the discipline tool, one of the authors largely recanted what they originally wrote in a subsequent publication in the *Huffington Post*,^37^ clarifying that their intention was to caution against inappropriate or punitive utilisation of time-out and acknowledging its strong evidence-base when employed appropriately, a stance that mirrors the results of this study.

Unfortunately, the original *Time* magazine article's enduring impact continues to shape the perceptions of many parents and a growing number of practitioners.^7,38^ An important implication for practitioners who oppose the use of time-out, whether because of having heard discredited or inaccurate research on time-out or simply because of their own negative feelings towards the method, is to re-visit the evidence for time-out and refocus on upholding the scientist–practitioner model of psychology. It is imperative for practitioners to critically evaluate the information they encounter to prevent the dissemination of harmful misinformation. Such misinformation not only prevents families from accessing evidence-based support but also has the potential to induce significant distress and guilt in the majority of parents who use time-out, fearing they may have unintentionally caused harm to their children. Instead, practitioners should guide parents in effectively implementing time-out and tailor the strategy as required for children who have experienced adversity,^39^ thereby enhancing the children's emotional well-being and strengthening their attachment bonds with caregivers.

It is important to note that the finding that inappropriate implementation was negatively associated with child mental health does not suggest that time-out use should be discouraged. Just like any therapeutic intervention, whether psychological, physical or medical, there is a potential for adverse effects or diminished benefits when not administered in line with established guidelines. These findings highlight the importance of evidence-based application of time-out, particularly within trauma contexts, to safeguard vulnerable children against harm and promote enhanced attachment security.

In conclusion, our findings align with decades-long research supporting the use of time-out as an appropriate and safe disciplinary tool associated with enhanced child well-being and support the use of appropriately implemented time-out among children with a history of adversity. A key challenge for the field now is to combat misinformation and to ensure evidence-based parameters of time-out are widely disseminated to practitioners and families in clinical and community settings.

## Supporting information

Roach et al. supplementary material Roach et al. supplementary material

## Data, analytic code and research material availability

The data, analytic codes and research materials that support the findings of this study are available on request from the corresponding author, A.R. The data are not publicly available owing to ethical restrictions.
