# Supplementary material for: Time-out under scrutiny: examining the relationships among the discipline strategy time-out, child well-being and attachment and exposure to adversity
Source: Br J Psychiatry. 2025 Aug;227(2):538–44. doi: 10.1192/bjp.2024.228 (PMC12355463; doi:10.1192/bjp.2024.228)
Supplement: Roach et al. supplementary material [file S0007125024002289sup001.docx]

**Supplementary material**

Table S1. Associations between Time-out and Adversity and Externalising Symptoms

|  | **Independent variable** | **B** | **95.0% CI for B** | ***p* Value** | **R^2^** | **Adjusted R^2^** |
| --- | --- | --- | --- | --- | --- | --- |
| Model 1 | Time-out | -.140 | -.198 - -.082 | <.001* | .157 | .153 |
|  | Adversity Exposure | .445 | .324 - .565 | <.001* |  |  |
| Model 2 | Time-out | .058 | -.006 - .122 | .077 | .403 | .393 |
|  | Adversity Exposure | .269 | .159 - .378 | <.001* |  |  |
|  | Parental Consistency | .119 | -.033 - .271 | .124 |  |  |
|  | Positive Encouragement | -.080 | -.286 - .126 | .444 |  |  |
|  | Parent Child Relationship | .202 | .050 - .355 | .010* |  |  |
|  | Coercive Parenting | .461 | .350 - .573 | <.001* |  |  |
|  | Family Relationships | .159 | .006 - .311 | .041* |  |  |
|  | Parental Adjustment | .137 | .004 - .270 | .044* |  |  |
| Model 3 | Time-out | .051 | -.014 - .115 | .125 | .408 | .396 |
|  | Adversity exposure | .316 | .196 - .436 | <.001* |  |  |
|  | Parental Consistency | .119 | -.032 - .270 | .123 |  |  |
|  | Positive Encouragement | -.064 | -.270 - .142 | .542 |  |  |
|  | Parent Child Relationship | .196 | .043 - .348 | .012* |  |  |
|  | Coercive Parenting | .474 | .362 - .586 | <.001* |  |  |
|  | Family Relationships | .148 | -.004 - .301 | .057 |  |  |
|  | Parental Adjustment | .131 | -.002 - .264 | .053 |  |  |
|  | ACE X TO | .013 | -.001 - .026 | .060 |  |  |

B = unstandardized regression coefficient; CI = confidence interval; ACE = Adverse Childhood Experiences; TO = Time-out

Model 1: Associations between Time-out and Adversity and Externalising Symptoms

Model 2: Model 1 + adjusted for parenting and family covariates

Model 3: Model 2 + interaction term (time-out x adversity)

*Significant *p < 0.05*

Table S2. Associations between Time-out and Adversity and Internalising Symptoms

|  | **Independent variable** | **B** | **95.0% CI for B** | ***p* Value** | **R^2^** | **Adjusted R^2^** |
| --- | --- | --- | --- | --- | --- | --- |
| Model 1 | Time-out | -.263 | -.316 - -.210 | <.001* | .287 | .284 |
|  | Adversity Exposure | .456 | .346 - .566 | <.001* |  |  |
| Model 2 | Time-out | -.089 | -.150 - -.028 | .004* | .454 | .445 |
|  | Adversity Exposure | .313 | .209 - .417 | <.001* |  |  |
|  | Parental Consistency | .162 | .018 - .306 | .028* |  |  |
|  | Positive Encouragement | -.191 | -.386 - .005 | .056 |  |  |
|  | Parent Child Relationship | .163 | .018 - .308 | .027* |  |  |
|  | Coercive Parenting | .327 | .221 - .433 | <.001* |  |  |
|  | Family Relationships | .218 | .073 - .363 | .003* |  |  |
|  | Parental Adjustment | .090 | -.037 - .216 | .165 |  |  |
| Model 3 | Time-out | -.100 | -.161 - -.039 | .001* | .464 | .454 |
|  | Adversity Exposure | .382 | .269 - .495 | <.001* |  |  |
|  | Parental Consistency | .162 | .019 - .305 | .026* |  |  |
|  | Positive Encouragement | -.167 | -.362 - .027 | .092 |  |  |
|  | Parent Child Relationship | .154 | .010 - .298 | .036* |  |  |
|  | Coercive Parenting | .345 | .240 - .451 | <.001* |  |  |
|  | Family Relationships | .203 | .059 - .347 | .006* |  |  |
|  | Parental Adjustment | .081 | -.044 - .207 | .204 |  |  |
|  | ACE X TO | .019 | .006 - .031 | .004* |  |  |

B = unstandardized regression coefficient; CI = confidence interval; ACE = Adverse Childhood Experiences; TO = Time-out

Model 1: Associations between Time-out and Adversity and Internalising Symptoms

Model 2: Model 1 + adjusted for parenting and family covariates

Model 3: Model 2 + interaction term (time-out x adversity)

*Significant *p < 0.05*

Table S3. Simple slopes analysis: Associations between Time-out and Internalising Symptoms for High and Low adversity

| **Independent variable** | **Moderator** | **B** | **95.0% CI for B** | ***p* Value** | **R^2^** | **Adjusted R^2^** |
| --- | --- | --- | --- | --- | --- | --- |
| Time-out | Low adversity | -.153 | -.227 - -.079 | <.001* | .464 | .454 |
|  | High Adversity | -.048 | -.114 - .019 | .161 |  |  |

Table S4. Associations between Time-out and Adversity and Insecure Attachment

|  | **Independent variable** | **B** | **95.0% CI for B** | ***p* Value** | **R^2^** | **Adjusted R^2^** |
| --- | --- | --- | --- | --- | --- | --- |
| Model 1 | Time-out | -.098 | -.110 - -.086 | <.001* | .358 | .355 |
|  | Adversity Exposure | .030 | .004 - .056 | .023* |  |  |
| Model 2 | Time-out | -.048 | -.061 - -.034 | <.001* | .545 | .537 |
|  | Adversity Exposure | .005 | -.018 - .028 | .679 |  |  |
|  | Parental Consistency | .035 | .003 - .068 | .032* |  |  |
|  | Positive Encouragement | -.037 | -.081 - .007 | .095 |  |  |
|  | Parent Child Relationship | .055 | .023 - .088 | <.001* |  |  |
|  | Coercive Parenting | .105 | .081 - .129 | <.001* |  |  |
|  | Family Relationships | .062 | .029 - .094 | <.001* |  |  |
|  | Parental Adjustment | -.032 | -.060 - -.003 | .028* |  |  |
| Model 3 | Time-out | -.046 | -.060 - -.032 | <.001* | .549 | .540 |
|  | Adversity Exposure | -.006 | -.032 - .020 | .649 |  |  |
|  | Parental Consistency | .035 | .003 - .068 | .032* |  |  |
|  | Positive Encouragement | -.041 | -.085 - .003 | .067 |  |  |
|  | Parent Child Relationship | .057 | .024 - .090 | <.001* |  |  |
|  | Coercive Parenting | .102 | .078 - .126 | <.001* |  |  |
|  | Family Relationships | .064 | .031 - .097 | <.001* |  |  |
|  | Parental Adjustment | -.031 | -.059 - -.002 | .035* |  |  |
|  | ACE X TO | -.003 | -.006 - .000 | .043* |  |  |

B = unstandardized regression coefficient; CI = confidence interval; ACE = Adverse Childhood Experiences; TO = Time-out

Model 1: Associations between Time-out and Adversity and Insecure attachment

Model 2: Model 1 + adjusted for parenting and family covariates

Model 3: Model 2 + interaction term (time-out x adversity)

*Significant *p < 0.05*

Table S5. Simple slopes analysis: Associations between Time-out and Insecure Attachment for High and Low adversity

| **Independent variable** | **Moderator** | **B** | **95.0% CI for B** | ***p* Value** | **R^2^** | **Adjusted R^2^** |
| --- | --- | --- | --- | --- | --- | --- |
| Time-out | Low adversity | -.038 | -.055 - -.021 | <.001* | .741 | .549 |
|  | High Adversity | -.054 | -.069 - -.039 | <.001* |  |  |

B = unstandardized regression coefficient; CI = confidence interval

*Significant *p < 0.05*

Table S6. Associations between Time-out and Adversity and Trauma Symptoms

|  | **Independent variable** | **B** | **95.0% CI for B** | ***p* Value** | **R^2^** | **Adjusted R^2^** |
| --- | --- | --- | --- | --- | --- | --- |
| Model 1 | Time-out | -.029 | -.039 - -.019 | <.001* | .215 | .211 |
|  | Adversity Exposure | .094 | .073 - .114 | <.001* |  |  |
| Model 2 | Time-out | -.014 | -.027 - -.001 | .032* | .253 | .240 |
|  | Adversity Exposure | .092 | .070 - .114 | <.001* |  |  |
|  | Parental Consistency | .012 | -.018 - .042 | .437 |  |  |
|  | Positive Encouragement | -.010 | -.052 - .031 | .617 |  |  |
|  | Parent Child Relationship | .006 | -.024 - .037 | .680 |  |  |
|  | Coercive Parenting | .047 | .025 - .070 | <.001* |  |  |
|  | Family Relationships | -.004 | -.034 - .027 | .810 |  |  |
|  | Parental Adjustment | -.025 | -.052 - .001 | .062 |  |  |
| Model 3 | Time-out | -.011 | -.024 - .001 | .079 | .270 | .256 |
|  | Adversity Exposure | .075 | .052 - .099 | <.001* |  |  |
|  | Parental Consistency | .012 | -.018 - .042 | .435 |  |  |
|  | Positive Encouragement | -.016 | -.057 - .025 | .439 |  |  |
|  | Parent Child Relationship | .009 | -.021 - .039 | .572 |  |  |
|  | Coercive Parenting | .043 | .021 - .065 | <.001* |  |  |
|  | Family Relationships | -2.602 | -.030 - .030 | .999 |  |  |
|  | Parental Adjustment | -.023 | -.050 - .003 | .082 |  |  |
|  | ACE X TO | -.004 | -.007 - -.002 | .001* |  |  |

B = unstandardized regression coefficient; CI = confidence interval

Model 1: Associations between Time-out and Adversity and Trauma Symptoms

Model 2: Model 1 + adjusted for parenting and family covariates

Model 3: Model 2 + interaction term (time-out x adversity)

*Significant *p < 0.05*

Table S7. Simple slopes analysis: Associations between Time-out and Trauma Symptoms for High and Low adversity

| **Independent variable** | **Moderator** | **B** | **95.0% CI for B** | ***p* Value** | **R^2^** | **Adjusted R^2^** |
| --- | --- | --- | --- | --- | --- | --- |
| Time-out | Low adversity | -.001 | -.016 - .014 | .885 | .269 | .256 |
|  | High Adversity | -.026 | -.039 - -.013 | <.001* |  |  |

B = unstandardized regression coefficient; CI = confidence interval

*Significant *p < 0.05*

Table S8. Correlations for study variables

|  | 1 | 2 | 3 | 4 | 5 | 6 | 7 | 8 |
| --- | --- | --- | --- | --- | --- | --- | --- | --- |
| 1. TO | 1.00 |  |  |  |  |  |  |  |
| 2. ALES | -.150^**^ | 1.00 |  |  |  |  |  |  |
| 3. Parental Consistency | -.561^**^ | .091^*^ | 1.00 |  |  |  |  |  |
| 4. Parent-child Relationship | -.347^**^ | .104^*^ | .374^**^ | 1.00 |  |  |  |  |
| 5. Positive Parenting | -0.01 | -0.04 | .099^*^ | .551^**^ | 1.00 |  |  |  |
| 6. Coercive parenting | -.470^**^ | .241^**^ | .454^**^ | .387^**^ | -0.01 | 1.00 |  |  |
| 7. Family Relationships | -.316^**^ | .220^**^ | .359^**^ | .488^**^ | .215^**^ | .457^**^ | 1.00 |  |
| 8. Parental Adjustment | -.093^*^ | .323^**^ | .254^**^ | .473^**^ | .268^**^ | .345^**^ | .539^**^ | 1.00 |
| **. Correlation is significant at the 0.01 level (2-tailed). | | | | | | | | |
| *. Correlation is significant at the 0.05 level (2-tailed). | | | | | | | | |
